# Society and personal genome data

**DOI:** 10.1093/hmg/ddy084

**Published:** 2018-03-07

**Authors:** Anna Middleton

**Affiliations:** 1 Society and Ethics Research Group, Connecting Science, Wellcome Genome Campus, Hinxton, Cambridge CB10 1SA, UK; 2 Faculty of Education, University of Cambridge, UK

## Abstract

Genomic data offer a goldmine of information for understanding the contribution of genetic variation makes to health and disease. The potential of genomic medicine, to predict, diagnose, manage and treat genetic disease, is underpinned by accurate variant interpretation. This in itself hinges on the ability to access large and varied genomic databases. There is now recognition that international collaboration between research and healthcare systems are paramount to delivering the scale of genomic data required. No single research group, institute or country will liberate our understanding, it is only through global cooperation, together with super computing power, will we truly make sense of how genotype and phenotype correlate. Whilst it is logistically possible to create computing systems that talk to each other and aggregate datasets ready to reveal novel correlations, the bottom line is that this will only happen if *people* (whether they be scientists, clinicians, patients, research participants, policy makers, politicians, law makers) support the principle that we should be donating, accessing and sharing our DNA data in this way. And in order to make the most sense of genomics, given the geographical and ancestral variation between us, such *people* are likely to be the majority of society. Within this review, a perspective is proffered on the human story that underpins genomic ‘big data’ access and how we are at a tipping point as a society—we need to decide collectively, are we in? and if so, what needs to be in place to protect us? or are we out?

## Introduction

According to the Chief Medical Officer to the United Kingdom (UK) government, ‘we need to welcome the genomic era and deliver the genomic dream!’ (1). Advances in genomic technology mean it is now cheaper and easier than ever before to analyse one or many of a person’s 20 000 genes, offering information to help diagnose, manage and treat genetic disease (2).

A defect in a single gene is thought to be the cause of the majority of ‘rare’ disease worldwide (3). By definition, this means that fewer than 5 people are affected per 10 000 (4), but as there are so many such conditions, collectively they are common (5). Estimates vary, but we think rare, single-gene conditions affect 1 in 17 of us (6).

Therefore, within our own social circles we are each likely to know (or be related to) someone with a serious, potentially life-threatening condition who could, in theory, receive benefit from a genomic test. Unlocking the molecular basis of rare disease will also be the key to our understanding and treatment of common diseases (7) and we should not underestimate the contribution that genomic^1^ knowledge makes to medicine in general (8).

Projects such as the Deciphering Developmental Disorders (DDD) study (9), offering exome sequencing to children with severe developmental disorders, report that if a clinical exome was offered as a first line diagnostic test, >50% of these children would instantly receive a diagnosis (10). With advances in genomic technology, where relevant, it should be possible to identify the prime genetic cause for every rare disorder. What underpinned the success of the DDD project was the ability to match children at opposite ends of the UK (and now the world) to each other, using a database called DECIPHER (11). As each child’s condition was uncommon—and for the doctors caring for that child, they may never have encountered a child with a similar condition before—the DECIPHER database afforded the opportunity to link children with the same genetic result and phenotype. This added to the credibility that the identified variant was indeed the cause of the child’s condition.

What typifies the success of genomic medicine is thus collaboration (5,7,12). An integrated systems approach is required—with computers and databases that talk to each other, we need people to enter accurate data into these databases, we need legal systems to enable safe, protected data sharing and we need patients and research participants to contribute their data for use by others. We also need policy recognition that spans borders, jurisdiction and countries, acknowledging that global cooperation is paramount.

## Genomic Medicine Depends on Large Genomic Datasets

The journey towards the translation of genomic technology into healthcare and the integration of genomic medicine within healthcare systems has definitely begun, indeed ‘genomics is not tomorrow, it is here today’ (1, p. 3). We have also reached a stage in history where Big Data and DNA go hand in hand (13).

The datasets required for variant interpretation involve genomic data from people of varying ages, ethnicities, differing stages of health and disease (14). There is global recognition that in order to avoid duplication of datasets and ensure quality assurance, data curation is paramount (15). We also need practical and ethical solutions for linking sequencing data with clinical phenotypes and networking computer systems to each other (14,16–18).

Genomic medicine is a discipline at the cutting edge of science, which relies heavily on input from both non-profit and for-profit research as well as participation with industry (19). Many of the databases that a clinical scientist will access in the NHS when doing genomic variant interpretation (e.g. DECIPHER or ExAC to list but two) are research databases. Within the scope of this review therefore, ‘genomic medicine’ blurs many boundaries between clinical and research practice. What is consistent, however, is that the evidence-base that guides genomic medicine is underpinned by the ability to consult large scale genomic datasets. And the data that sits in these databases at one point in time belonged to a person who chose (by virtue of a consent process or by condition of access to a health service) for their de-identified data to be donated and accessed by others.

## DNA and Big Data

The sharing of generic health data within/between healthcare systems and research has already been happening for many years—and is integral to healthcare evaluation. The concept of health data sharing is also, broadly speaking, acceptable to the public (20–23). However, we are now entering a new era of connectivity, with plans to link entire health systems, across countries, to each other (24); together with recognition of the importance of collaborating between non-profit and for-profit industries (25).

According to the European Commissioner for Health and Food Safety at the European Parliament: ‘Big data has enormous potential to advance medical research, bring about greater innovation in healthcare, and improve the overall performance of health systems’ (26, p. 1). The Million European Genomes Alliance (MEGA) aims to be operating by 2020, whereby existing and new research and clinical sequencing programmes connect and share data, crossing geographical borders and legal jurisdictions (24).

Every time a citizen uses a hospital service or participates in a biobank or research project, there is a new opportunity for a fresh set of health data to join the grid. The issue of data donation, access and sharing now appears to be relevant to more of us than ever before—even if we are not personally being asked to donate our data, we might be genetically related to someone who is, and so the decisions they make could be pertinent to us too (27).

The acceptance that one’s personal health data will be used by others in the research endeavour is usually predicated on the condition that the data is ‘anonymous’^2^ and that careful steps have been taken to protect the donor’s privacy. And yet, our genome is our most personal and unique identifier. Unless we have an identical twin, our sequence is the barcode that identifies us as ‘us’. Even if only electronic sections of our genome are stored and shared, if we have something particularly rare, then there is always the risk that we can be identified from this alone (18,28–30). This becomes especially relevant if we have other information about ourselves connected to the genomic data (e.g. an unusual clinical characteristic) and also if we have other information in the public domain (e.g. our name appears in an ancestry database or we provide details about our health on Facebook). We must always assume, therefore, that there is a possibility we could be identified if our genomic data sits in a database.

Genomic data is traded, collated, re-categorized and literally bounced around the Internet every second of the day (18); when it has been accessed once and shared with others, it may be difficult to retract (18). Thus, it is negligent to imply that it would be easy or straightforward (or even possible) to withdraw an individuals’ genomic data from every imprint on the Internet once it has been deposited and made available for access.

What will help to reassure us that the pros of data sharing outweigh the cons? ‘People need to be satisfied that genomic medicine operates in their common interests, whilst protecting their individual privacy, and does not exploit some to benefit others. Protection of individual privacy cannot be absolute, nor can data ever be guaranteed as entirely secure, but there needs to be an understanding of the associated risks and reassurance that breaches are appropriately prosecuted’ (31, Chap. 16, p. 4).

## Security Breaches

Policy makers and scholars alike recognise that publics^3^ and patients, need to know that their data is protected, safe and won’t lead to personal harm (32). Shabani and Borry point out ‘Adopting adequate privacy protections for genomic data has been endorsed by the establishment of the International Declaration on Human Genetic Data, which was issued…by UNESCO as complementary to its Universal Declaration on Human Genome and Human Rights’ (33). Thus, as a principle, we should not be discriminated against, on the basis of our genomic information, for example, by employers or insurance companies. This latter point has been subject of considerable debate internationally (34) and indeed in many jurisdictions there are legal protections in place (35). Nevertheless, the *perception* and *fear* of discrimination is significant amongst many publics and so clearer messaging around this would be helpful.

Of particular interest in Europe is the application of the new General Data Protection Regulation and how it will be applied to genetic data (33,36). Here, significant fines will be imposed on the person/organization in control of the data if there is a security breach and individuals are identified.

What harm can actually come of being identified? In reality, ‘identification’ whilst undesirable, would likely consist of a name and/or other personal identifiers connected to a stretch of DNA code. On its own, and without access to specialist bioinformatics software, no clinical inferences are obvious. However, the personal stigma attached to this becomes apparent if clinical or health data are also revealed at the same time. But again, what actual harm can come of this? The harms can become reality, not from the data being known, per say, but from someone *doing* something with that data. In a court of law a claimant would have to provide tangible evidence of financial, or reputational damage in order to be able to sue for damages; ‘embarrassment’ on its own, is unlikely to be sufficient.

Application of the GDPR and case law on data security breaches will lead to further clarification on the legal responses to being identified. In the meantime, the Parliamentary Under-Secretary of State for Health published the UK government’s response to the Caldicott review and concluded that ‘Boosting cyber resilience, improving the response to data and cyber incidents and providing clarity on the handling of personal data remain an urgent priority for the health and care sector’ (37).

## We Need Empirical Attitude Data from Publics

In order to focus collective efforts on finding out what harms can come, if we are identified from our genomic data, we can usefully ask global publics what they would like to be protected from. Large scale attitude studies are needed, both quantitative and qualitative in method, to explore, from many different angles, what we as a society fear from being identified. Such studies are also useful for gauging the temperature on whether assumptions made in policy (e.g. that we should all donate our data for use in research) are likely to receive a public backlash—and we should never underestimate the power of public dissent to policy rolled out without proper consultation (38).

What is striking from the research that has been done to date on public attitudes is that people are not averse to contributing to research and participants feel a sense of being part of a ‘public good’ (39, 40), but they become understandably agitated when they are not consulted on the uses of their data (34) or are left with suspicions their contribution will lead to profits for big corporations or companies (41). Research from, for example from Wellcome has shown that when time is taken to fully explain the necessity for a partnership between industry and healthcare and that medicines won’t be developed without the cooperation of the pharmaceutical industry, publics are more accepting of their data being used in the for-profit industry (42).

## Something in Return?

In recognition of the value that individual genomes have in the research endeavour, there have been calls for reciprocity, i.e. giving something back in return for data donation. This could be cash or individual results or raw data (43,44). The ethical issues relating to this are subject to increasing debate (45,46).

## Assumed Altruism

The UN Declaration of Human Rights informs us that collectively we all have the right to benefit from science (47). But to benefit from science, in return we should contribute to science (48). Within the patient support groups for people with genetic conditions, we are encouraged to share our data ‘it is important to gather information from as many patients as possible’ (Genetic Alliance UK website on ‘What is patient data and how is it used?’); we are also told that genomic data sharing is there for the ‘global good’ (14) and genomic data sharing is about ‘all of us’ (49).

In the 100 000 Genomes Projects, participants do not have a choice in this—participation in the project means that patient data *will* be accessed and shared with others (50). Thus, within the genomics world, assumptions are already being made about the ‘social contract’ (48): ‘A commitment to open access, global genomic data and knowledge sharing reflects a call for a new social contract based on a principle of solidarity and a duty to act for the common good’ (48). This implies that if we expect excellence in our healthcare then we should all be prepared for our data to be used within the evidence base that underpins that healthcare.

Particularly within research, a condition of participation is often that research data (which may include raw sequence data for example) will be deposited in a repository for secondary analysis by others (51,52). However, this brings ethical tensions as it is assumed that people *should* be altruistic with their contribution, drawing on the literature surrounding those who decline genetic testing shows us that people who choose to opt out of testing or showed dissent were seen by their family as difficult or ‘lacking in character’ (53). This suggests that when there is pressure to be altruistic, declining participation, may be difficult.

## Socializing the Benefits of Genomics for Society

What would help here are clear explanations as to the risks and benefits of data donation, access and subsequent sharing. Plus, a global public engagement strategy delivered at a high enough level to have an international reach. It is insufficient to rely on the charitable sector, individual healthcare systems, research projects or even funding bodies to deliver this strategy. Involvement of the World Health Organization and United Nations may be a good start. Whilst it is completely unrealistic to believe that we should achieve some sort of public ‘world consensus’ on the principle of data donation and sharing, before we can continue, we should at least bring the concepts of a collective altruism into societal discussion and social consciousness. Despite calls for ‘public education’ in genetics (54), work in the science communication and public engagement literature suggests publics respond to more social and sociable ways of engagement (55) to increase their science capital (56).

At the heart of any future engagement about genomic data access needs to be an exploration of trust (38,57,58), together with a clear explanation of the legal sanctions in place to protect us. We need to feel reassured that our contribution will bring no personal harm to us or our relatives and that we need to understand how our data will contribute to understanding human health. We also need to feel that we have a choice about participation.

## Global Alliance for Genomics and health

The Global Alliance for Genomics and Health (GA4GH) is a non-profit organization that unites >500 healthcare providers, universities, research institutes, funders, patient advocacy groups and industry all with the ‘shared ideal that sees maximizing the public good as a chief priority of genomic innovation in health’ (59). Guided by Human Rights legislation (60) the GA4GH Framework ‘provides guidance for the responsible sharing of human genomic and health-related data, including personal health data and other types of data that may have predictive power in relation to health…[and is based on]… human rights of privacy, non-discrimination and procedural fairness’ (60).

Historically, researchers have very unhelpfully worked in silos (61), with a resistance to share their data for use by other researchers. Other researchers have wanted to share their data but have had concerns about how best to do this ethically (18). There is now agreement (52,62) that a parochial way of working, which also applies to industry (63), should not be tolerated. Indeed, many funders now require their researchers, as a condition of funding, to deposit raw research data into repositories to that it can be opened up for secondary analysis (52,64). The GA4GH aims to provide the ethical and regulatory infrastructure, IT solutions and practical support to enable data to be shared globally so that we as a society feel comfortable and protected that our altruism is indeed beneficial to others as opposed to harmful to ourselves. 

## Conclusion

Genomic medicine involves the analysis of a person’s genome and the use of this to inform diagnosis, disease management and, where possible, personalized treatment, tailor-made to the individual’s germline and/or somatic genomic profile. This is the vision. And yet, ‘To realise the full impact of genomic medicine, genomic and clinical data must be interoperable across traditional geographic, jurisdictional, sectoral and domain boundaries. Extremely large and diverse data sets are needed to provide a context for interpretation of genetic sequences. No single country or institution can achieve the necessary scale and diversity alone. Data must be shared’ (65, p. 104).

Whilst it is logistically possible to create computing systems that talk to each other and aggregate datasets ready to reveal novel correlations, the bottom line is that this will only happen if *people* (whether they be scientists, clinicians, patients, research participants, policy makers, politicians, law makers) support the principle that we should be donating, accessing and sharing our DNA data in this way. And in order to make the most sense of genomics, given the geographical and ancestral variation between us, such *people* need to be most of us. It is thus time for global leadership to enable us as a society to be part of the conversation about the use of our data. We need the concepts of genomic data access to be socialized for us so that we can trust that our participation will not harm us but moreover will be good for us and society. We also need a collective buy to the concept of altruism. All of us need to decide, are we in or are we out?

## Footnotes

1 Terminology is used very broadly in this review, e.g. ‘genomic’ could equally refer to either germline or somatic genomic data. ‘Genetic’ and ‘genomic’ both refer to the ability to search through DNA to understand and catalogue variation, when only one gene is explored ‘genetic’ is used, when many genes are explored then ‘genomics’ is used. We do not specify lab techniques on offer, but they may include, e.g. WGS, WES, microarray, SNP based testing etc. ‘Genomic medicine’ covers the healthcare discipline using genomic testing to enhance patient care; however, ‘genomic medicine’ also includes within it a backdrop of basic as well as clinical (both non-profit and for-profit) research that offer the pivotal datasets consulted in variant interpretation.

2 The terms used to describe the varying levels of anonymity are inconsistent (pseudonomised, de-identified, de-linked might be used interchangeably); very helpful work from the Global Alliance for Genomics and Health provide a clear lexicon of definitions.

3 We use the terms ‘publics’ instead of ‘pubic’ to respect that we (public) are not a singular, homogenous group of people, but a diverse mixture of many individuals and communities.
